# Nutrition, Diabetes and Tuberculosis in the Epidemiological Transition

**DOI:** 10.1371/journal.pone.0021161

**Published:** 2011-06-21

**Authors:** Christopher Dye, Bernadette Bourdin Trunz, Knut Lönnroth, Gojka Roglic, Brian G. Williams

**Affiliations:** 1 HIV/AIDS, Tuberculosis, Malaria and Neglected Tropical Diseases, World Health Organization, Geneva, Switzerland; 2 Petit-Lancy, Geneva, Switzerland; 3 Stop TB Department, World Health Organization, Geneva, Switzerland; 4 Chronic Diseases and Health Promotion, World Health Organization, Geneva, Switzerland; 5 South African Centre for Epidemiological Modelling and Analysis (SACEMA), Stellenbosch, South Africa; Swiss Tropical and Public Health Institute, Switzerland

## Abstract

**Background:**

Diabetes prevalence and body mass index reflect the nutritional profile of populations but have opposing effects on tuberculosis risk. Interactions between diabetes and BMI could help or hinder TB control in growing, aging, urbanizing populations.

**Methods and Findings:**

We compiled data describing temporal changes in BMI, diabetes prevalence and population age structure in rural and urban areas for men and women in countries with high (India) and low (Rep. Korea) TB burdens. Using published data on the risks of TB associated with these factors, we calculated expected changes in TB incidence between 1998 and 2008. In India, TB incidence cases would have increased (28% from 1.7 m to 2.1 m) faster than population size (22%) because of adverse effects of aging, urbanization, changing BMI and rising diabetes prevalence, generating an increase in TB incidence per capita of 5.5% in 10 years. In India, general nutritional improvements were offset by a fall in BMI among the majority of men who live in rural areas. The growing prevalence of diabetes in India increased the annual number of TB cases in people with diabetes by 46% between 1998 and 2008. In Korea, by contrast, the number of TB cases increased more slowly (6.1% from 40,200 to 42,800) than population size (14%) because of positive effects of urbanization, increasing BMI and falling diabetes prevalence. Consequently, TB incidence per capita fell by 7.8% in 10 years. Rapid population aging was the most significant adverse effect in Korea.

**Conclusions:**

Nutritional and demographic changes had stronger adverse effects on TB in high-incidence India than in lower-incidence Korea. The unfavourable effects in both countries can be overcome by early drug treatment but, if left unchecked, could lead to an accelerating rise in TB incidence. The prevention and management of risk factors for TB would reinforce TB control by chemotherapy.

## Introduction

Although most countries with a high burden of tuberculosis (TB) have adopted and widely implemented the World Health Organization's Stop TB Strategy, the rate of decline in case numbers has been slower than expected [1], [2]. Possible explanations include patient and health system delays in diagnosis and treatment, and the rise of risk factors including co-infections (notably with human immunodeficiency virus, HIV), air pollution, alcohol abuse, crowding, diabetes, malnutrition, tobacco smoking and urbanization [3].

Low body mass and diabetes have been treated as distinct risk factors for tuberculosis [4], [5], [6], [7] although they are linked components of the nutritional profile of populations. While diabetes enhances the risk of pulmonary TB [4], [8], [9], [10], a greater body mass index (BMI) is protective [6], and yet diabetes is more frequent among people who are overweight [11], [12], [13]. To add to the complexity at population level, BMI distribution, diabetes prevalence and TB incidence vary by age and sex and differ between rural and urban areas. In particular, TB incidence changes with age directly (because the prevalence of infection and the risk of progression from infection to active TB are age-dependent), and indirectly through its effects on BMI and DM as risk factors. Population aging is expected to affect TB incidence through these direct and indirect routes. The same is true of urbanization. This web of interactions raises the question of how TB incidence is likely to change as countries proceed through the epidemiological transition. Will TB control programmes be helped or hindered as diabetes prevalence increases with better nutrition in growing, aging, urbanizing populations?

This study examined the consequences for TB epidemiology and control of changes in BMI, diabetes, population age structure and urbanization in two contrasting countries for which there are substantial bodies of data: India, which is in a comparatively early stage of epidemiologic and demographic transition, has a high burden of TB per capita and an increasing prevalence of diabetes; and the Republic of Korea (hereafter Korea), which is at a later stage of transition, has a lower TB burden, and a stable or declining prevalence of diabetes. Our goal is not to estimate and explain the actual changes in TB incidence over time (the period 1998–2008) but rather to evaluate the effects of these specific nutritional and demographic factors as they reinforce or oppose other processes. Important among the other processes is TB control by chemotherapy, which we consider in the final discussion.

## Methods

We compiled data that describe how BMI, diabetes prevalence and population age structure in rural and urban areas changed through time for adult men and women in India and Korea. We used published information on the risks of TB associated with BMI, diabetes and urbanization, by age and sex, to calculate expected changes in TB incidence attributable to these factors, with all others assumed constant, between 1998 and 2008. We examined the effects of all factors jointly, and then separately to investigate which had the largest effects, and how these effects differed between countries. Our calculations did not include changes in TB case detection and treatment outcomes because the aim was to explore the effects of nutritional and demographic factors independent of the performance of TB control programmes.

### Modelling interacting risk factors for TB

We defined the interactive effect of BMI and diabetes on tuberculosis incidence per person per year, *I_i_*, in any group of people, *i*, by:
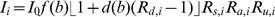
(1)


This expression is derived by assuming that *I_i_* is inversely related to BMI (abbreviated to *b*) via function *f*(*b*), while the prevalence of diabetes, *d*(*b*), increases with BMI. Function *d*(*b*) is defined by data as described below. We have previously shown [6] that function *f(b)* generally takes the form:
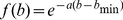
(2)


Among people who do not have diabetes, the per capita incidence of TB is *I_0_ f*(*b*). Among people with diabetes TB incidence is augmented by relative incidence, *R_d_*, which depends on age. With or without diabetes, the TB incidence rate also depends on sex (relative incidence, *R_s_*, is higher for men than women), age (*R_a_* is greater in older than younger people in India and Korea) and whether the group lives in an urban or rural area (*R_u_*). Thus equation (1) defines how, in general, TB incidence falls in better nourished populations (as measured by BMI), but increases among overweight people who develop diabetes. It also allows for the effects of aging and urbanization on TB incidence, directly though the age-dependent prevalence of infection and rate of progression from infection to disease, and indirectly through BMI and diabetes as TB risk factors. We have not allowed for other possible interactions between variables because they are not supported by data. For example, we have assumed that the relative risk of TB among people with diabetes changes with age in the same way for men and women (Table 1).

**Table 1:** Variables used to calculate changes in TB incidence with model (1).

Variable	India	Source	Korea	Source
**Differences between years**				
Years compared	1998, 2005		1998, 2008	
Adult (≥15 years) population size in initial, final years (millions)	655.2, 764.8	[16]	32.5, 37.3	[16]
Average age of adult population in initial, final years	36.5, 37.1	[16]	39.1, 42.6	[16]
Proportion adults living in urban areas in initial, final years	0.136, 0.148	[17]	0.395, 0.407	[17]
Average BMI in initial, final years (kg/m^2^)	23.7, 23.6	[18], [35]	23.2, 23.6	[20], [21], [22]
Diabetes prevalence in initial, final years	0.030, 0.037	[23], [26]	0.098, 0.087	[20], [21], [22]
Estimated TB cases/100,000 adults in 1998 (2.5^th^, 97.5^th^ centiles)	240 (211, 316)	[36]	124 (108, 140)	[36]
**Differences among age groups**				
Adult age groups (lower limits)	15, 50		20, 40, 60	
Proportion of adults in each age class in initial, final years	0.798, 0.2020.782, 0.218	[16]	0.514, 0.325, 0.1610.406, 0.383, 0.211	[16]
Relative prevalence (sd) of diabetes in older vs younger adults (lowest group = 1)	2.41 (0.08)	[24], [25], [37]	3.23 (0.20), 5.81 (0.35)	[20], [21], [22]
Relative incidence (sd) of TB among people with diabetes, by age, *R_d_*	4.03 (0.46), 2.68 (0.27)	[10], [38]	3.90 (1.23), 3.31 (0.29), 2.08 (0.51)	[38]
Relative incidence of TB by age, *R_a_* (lowest group = 1)	1.19	[36]	1.07, 2.62	[36]
**Differences among BMI groups**				
Body Mass Index, BMI groups (lower limits)	<18.5, 18.5, 25, 30	[18], [35]	<18.5, 18.5, 22, 23, 24, 25, 26, 30	[20], [21], [22]
Relative prevalence (sd) of diabetes by BMI group (lowest group = 1)	1.1 (0.40), 1.8 (0.27), 2.8 (0.22)	[23]	1.29 (0.07), 1.47 (0.05), 1.59 (0.05), 1.58 (0.05), 1.48 (0.05), 1.76 (0.06), 1.71 (0.09)	[20], [21], [22]
Rate of change of per capita TB incidence with BMI, α (sd)	−0.138 (0.002)	[6]	−0.138 (0.002)	[6]
**Differences between sexes**				
Relative incidence of TB among men vs women, *R_s_* (women = 1)	2.14	[2]	2.18	[2]
**Differences between urban and rural areas**				
Relative prevalence (sd) of diabetes, *d* (rural = 1)	2.03 (0.58)	[24], [25], [37]	1.24 (0.76)	[27]
Relative incidence (sd) of TB, *R_u_* (rural = 1)	1.69 (0.40)	[14]	0.48 (0.1)	[15]

Errors attached to estimates are standard deviations (sd), which are used in uncertainty analysis.

*R_u_* is higher in urban than rural areas in India, as inferred from measurements of the annual risk of infection [14]; the reverse is true in Korea, based on population-based surveys of disease prevalence [15]. For India, data are available for four BMI groups, both sexes, two age classes, and for urban and rural areas, making *I* = 32 groups in total. For Korea, there are data for eight BMI groups, both sexes, three age classes, and for urban and rural areas, making *I* = 96 groups in total. The incidence rate, *I*, in the whole population of size *N* in any year is the weighted average across all 32 or 96 groups, 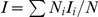.

Model (1) was fitted to data (below) for the initial year, 1998, by adjusting *I_0_*, so as to obtain a target value of *I* in that year (the WHO estimate) [2]. We then entered measured values of parameters and variables for the final year (2005 for India and 2008 for Korea) to calculate the expected change in TB cases (total number and per capita) and in the number of TB cases among people with diabetes. We also entered the data for each factor separately to calculate the contribution of each to overall changes in TB incidence. The calculated effects for India were adjusted to cover the period 1998–2008 in order to make comparisons with Korea over the same decade. Point estimates of the changes in TB incidence are accompanied by a Monte Carlo estimate of the 95% confidence limits, obtained by carrying out 1000 simulations by Latin Hypercube sampling of normally distributed variables in @Risk (Palisade Corporation).

Model (1) does not allow for changes in *Mycobacterium tuberculosis* transmission and TB incidence that would follow from nutritional and demographic changes between 1998 and 2008, the implications of which are discussed at the end of the paper.

### Sources of data

**Figure 1:**
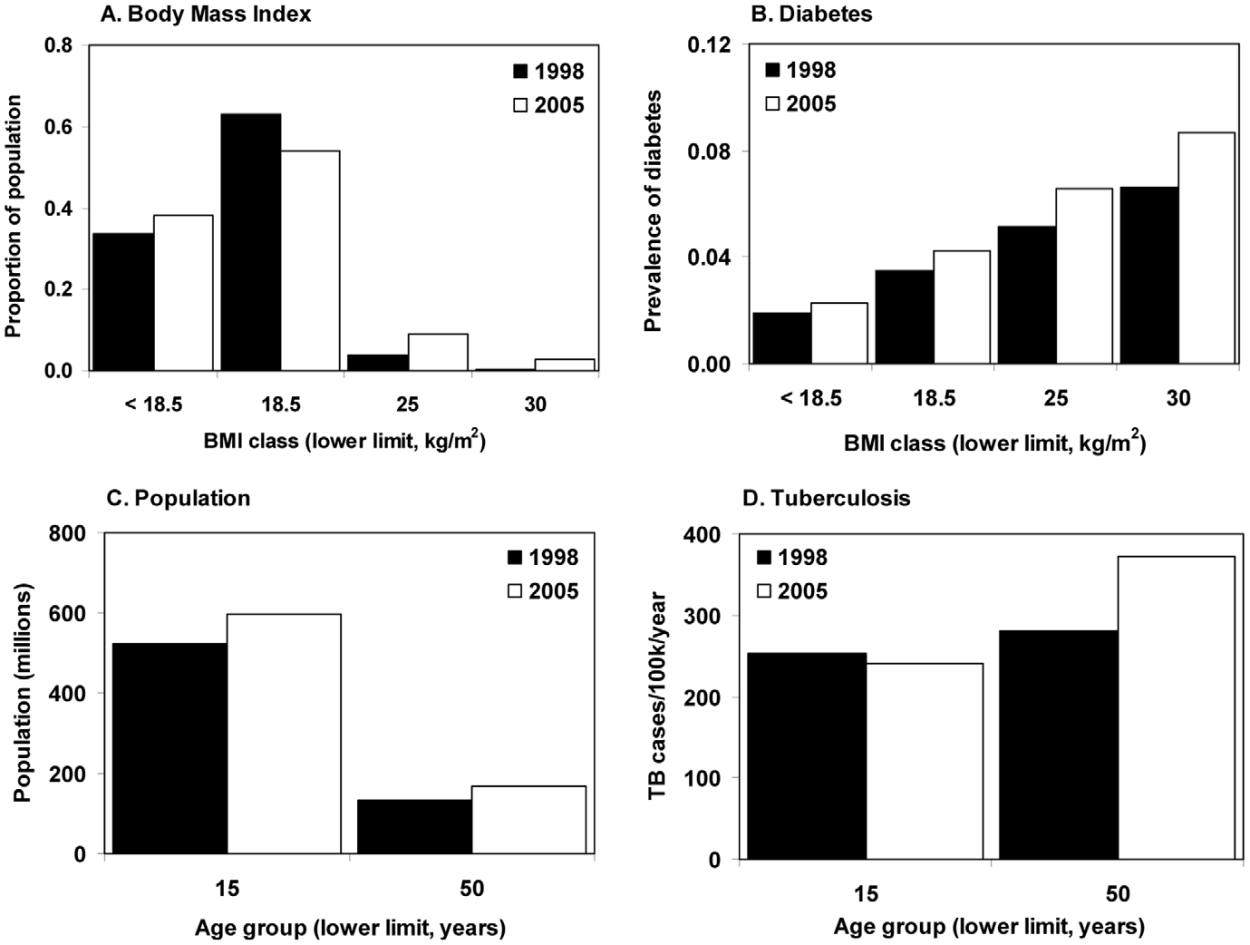
Distribution of (A) population and (B) diabetes prevalence by BMI group, and (C) population and (D) new TB cases per 100,000 population in each age group in India in 1998 (filled bars) and 2005 (open bars).

The data needed to satisfy model (1) are listed in table 1 and illustrated in figures 1 (India) and 2 (Korea). Further details are in Text S1.

**Figure 2:**
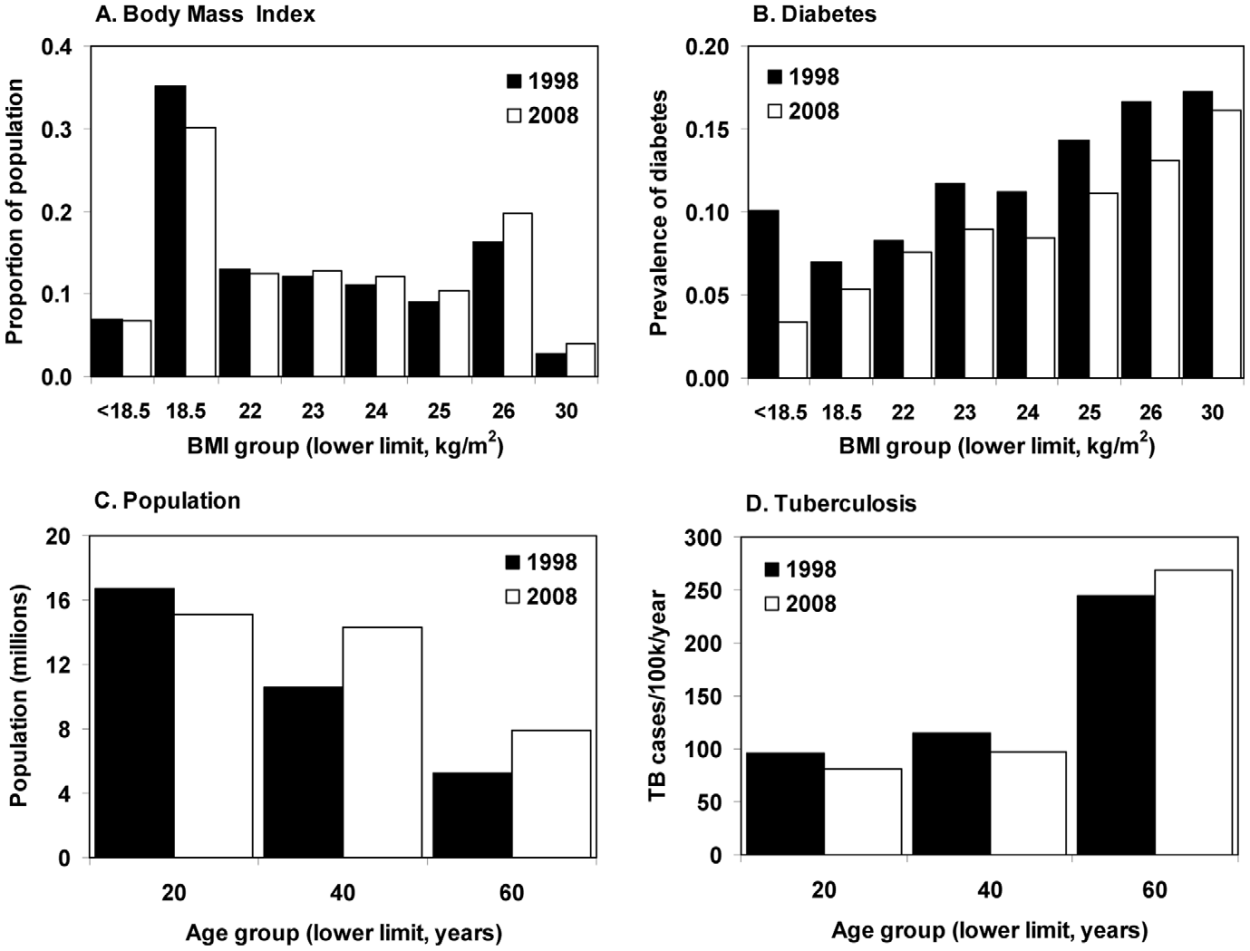
Distribution of (A) BMI, (B) diabetes prevalence, and (C) population and (D) new TB cases per 100,000 population in each age group in Korea in 1998 (filled bars) and 2008 (open bars).

#### Populations

Numbers of people by age, sex and year, and in urban and rural areas, are from the United Nations Population Division [16], [17]. This source gives point estimates with no uncertainty in numbers, rates of increase or population age structure.

#### Body Mass Index

The principal sources of data for both countries are national, population-based health surveys. For India, the distribution of BMI by age and sex, and for urban and rural areas, is reported in National Family Health Surveys for 1998–99 (NFHS-2) [18] and 2005–06 (NFHS-3) [19]. We take the reference years to be 1998 and 2005. Similar data for Korea come from National Health and Nutrition Examination Surveys (KNHANES) [20], [21], [22]. Five surveys were carried out between 1998 and 2008. We used data from the first and last surveys, checking that trends were consistent with data from the three intermediate surveys (2001, 2005 and 2007). Standard deviations of the proportions of people in each group, *i* (age, sex, rural/urban, year), used in uncertainty analysis, were calculated from sample sizes given in the survey reports.

#### Diabetes

For India, the main source of data was the Prevalence of Diabetes in India Survey 1999–2002 (PODIS) [23], a random multistage cross-sectional population survey, which reported diabetes prevalence by BMI, age, sex and in urban and rural areas. We checked the consistency of data with two other surveys carried out in India in 2000 (stratified random sampling, confined to urban areas) [24] and 2002 (self-reported diabetes in selected urban and rural centres) [25]. The national trend in diabetes was derived from the only site in India (Chennai) where four surveys carried out between 1998 and 2004 used similar methods [26]. For Korea, KNHANES surveys give diabetes prevalence by age, sex and BMI [20], [21], [22]. In addition, a 2006 survey in the south-east of the country reported differences between urban and rural areas [27]. Allowing for the observed differences by age, sex and between urban and rural areas, diabetes prevalence was scaled to generate the estimated national averages in initial and final years in both countries.

#### Tuberculosis

Target values of *I* are WHO estimates [2] of the incidence per capita of all new cases of pulmonary and extrapulmonary TB in 1998, assuming that all cases occur in the adult population (≥15 years and ≥20 years in India and Korea, respectively). Differences in per capita TB incidence by age and sex were derived from routine reports of sputum smear-positive cases, and applied to all forms of TB [2]. Compared with Korea, India is at an earlier stage of the epidemiological and demographic transition: India has a younger and faster growing population; TB incidence per capita is higher and cases are on average younger; the prevalence of diabetes is lower; and a higher proportion of the population lives in rural areas.

## Results

### India

**Figure 3:**
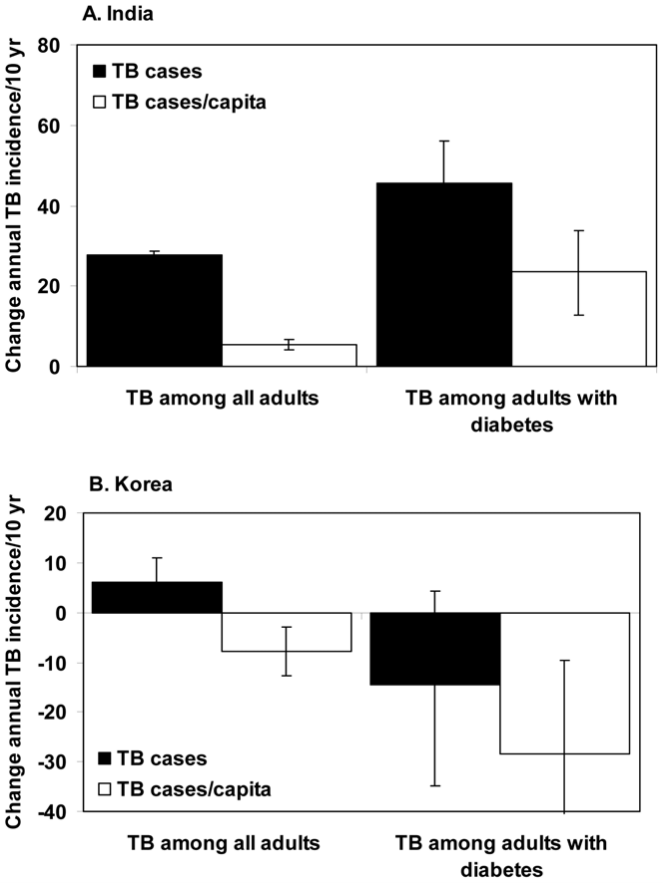
The net effects of nutritional and demographic changes on TB and TB among people with diabetes in (A) India and (B) Korea, expressed as the change over 10 years (1998–2008) in annual incidence (filled bars) and annual incidence per capita (open bars). Errors bars are 95%CL.

**Figure 4:**
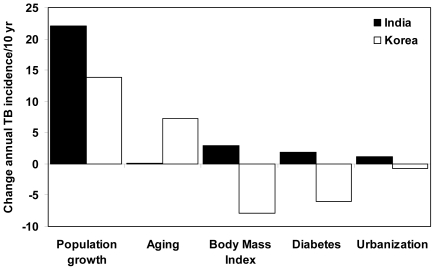
Changes in the annual number of new TB cases between 1998 and 2008 in India (filled bars) and Korea (open bars) attributable to each of five factors (horizontal axis) acting separately.

The number of new TB cases in India would have increased by 28% (95%CL 26–29%) from 1.73 million in 1998 to 2.10 million in 2008, faster than population growth (22%), because of the adverse effects of changing BMI, rising diabetes prevalence, urbanization and aging, in that order of importance (figures 1, 3, 4). The difference between the growth in population and in the number of TB cases gives an increase in TB incidence per capita of 5.5% (4.3–6.8%) in 10 years. That is, all the nutritional and demographic factors under investigation here would have increased TB incidence, in total and per capita, if all other factors had remained constant between 1998 and 2008.

Although the increase in BMI in India's urban population, and in women in rural areas, would have reduced TB incidence per capita, that positive effect was offset by a fall in BMI (from 21.3 to 20.9 kg/m^2^) among the majority of men who still live in rural areas (85% in 2005), and among whom TB incidence per capita is more than twice as high as in women (table 1). These changes in BMI would have caused a 2.9% increase in annual TB incidence between 1998 and 2008 (figure 4).

The prevalence of diabetes in India increased from an estimated 3.0% to 3.7% over the period of study. Calculations with model (1) indicate that most of the 0.7% increase (i.e. 0.6%) can be explained by changes in BMI and aging; the rest would be due to factors not included in this analysis. This rise in diabetes prevalence would have increased the annual number of TB cases in people with diabetes by 46% (35–56%) from 163,000 in 1998 to 224,000 in 2008, and the corresponding TB incidence per person with diabetes by 24% (13–34%) (figure 3). This amounts to an extra 0.90 million (0.50–1.33 million) TB cases among people with diabetes between 1998 and 2008.

Urbanization tends to push up TB incidence in India because the annual risk of infection has been found to be 69% higher in urban than rural areas (table 1). In this analysis, urbanization in India generates more TB cases because both transmission and the prevalence of diabetes are measurably higher in urban than rural areas. However, urbanization is a weaker force than BMI and diabetes because the proportion of people living in urban areas increased by only 1.2% (from 13.6% to 14.8%) between 1998 and 2005 (figure 4).

Aging of the Indian population augments the effect of net population growth because growth is higher in older age groups (29% in people ≥50 years, compared with 22% in all adults ≥15 years) that have higher TB incidence rates. However, population aging is the weakest of the adverse effects on TB in India (figure 4).

### Korea

In contrast with India, Korea's population has been growing more slowly (14%) and aging more quickly, so that a higher proportion of people moved into older age groups where TB incidence per capita is significantly greater (figure 2). Population growth, with a disproportionate increase in elderly people, contributed to a 6% (1–11%) rise in the number of TB cases from 40,200 to 42,800 between 1998 and 2008 (figure 3). The increase in number of people, especially older people (in an aging population), was the only adverse effect on the TB trend in the Korean population (figure 4).

The positive effects of trends in BMI, diabetes and urbanization would together have generated an 8% (3–13%) drop in TB incidence per capita in 10 years. Unlike India, BMI has been increasing in both men and women living in urban and rural areas, reducing TB incidence in all four groups, and therefore in the whole population (figure 4). TB incidence per capita is lower in urban than rural Korea (table 1), so TB incidence tends to fall as a growing proportion of people lives in urban areas.

Diabetes prevalence in Korea was about three times higher than in India over the period of study (table 1) but, in contrast with India, the prevalence was stable or falling, and so therefore were the numbers of TB cases among people with diabetes (figures 3, 4). An additional mitigating factor is that the prevalence of diabetes is not much higher in urban than rural areas (table 1), so the problem of TB linked to diabetes is little affected by urbanization.

## Discussion

The dual burden of infectious and non-infectious diseases [28] is not only coincidental but interactive, and the direction and magnitude of effects depend on accompanying demographic and environmental changes. TB incidence was falling in the industrialized world in the pre-chemotherapy era (at ∼5% per year), and social and economic development reinforced the effect of drugs when they became available in the 1950s [29]. Our study shows quantitatively how five specific, interacting, developmental factors – population growth, aging, body mass index, diabetes and urbanization, are working for and against contemporary TB control programmes in two contrasting Asian countries. We find that the combination of nutritional and demographic changes operating over the decade from 1998 tended to increase TB incidence per capita in high-burden India and reduce it in lower-burden Korea.

As the Indian population increased in size between 1998 and 2008, growth was relatively high in older people among whom TB incidence is higher. Urbanization has added to the problem because the annual risk of infection is higher on average in towns and cities [14]. However, the strongest adverse effect on TB incidence per capita in India was the fall in BMI among men living in rural areas. Concurrent under- and over-nutrition is a widespread problem in developing countries [30]; in that context, TB incidence in India would have increased both because of falling BMI in one part of the population and because of the overall rise in diabetes prevalence. Whether the fall in BMI among rural men was coincident with a rise in diabetes is not known because no surveys have measured trends in BMI and diabetes in the same rural population.

Although Korea has a lower TB incidence per capita than India – the result of four decades of rapid decline towards the end of the 20^th^ century [15] –incidence has now stabilized at a level that is substantially higher than in all other OECD countries (estimated at 88/100,000, in the whole population in 2008 [31]). Part of the reason is that, while the population size of Korea grew more slowly than that of India, the Korean population has been aging more quickly. The number of people over 50 years grew by 38% between 1998 and 2008; that is, population growth was highest in the older age groups that have a relatively high prevalence of infection and produce most TB cases per capita (table 1). Despite these negative effects of aging, our calculations indicate that increasing BMI, plus stable or falling diabetes prevalence, would have maintained a slow decline in TB incidence per capita in Korea, even if there was a small increase in the total number of cases.

Although nutritional and demographic changes have had stronger adverse effects in India than Korea, the magnitude of the effects in both countries was small compared to the potential for control by chemotherapy. Incremental improvements in TB case detection and cure have the potential to reduce incidence by 5–10% per year, and by 40–60% over a decade [32], [33]. For a given fraction of cases detected and cured, the decline tends to be faster where a higher proportion of cases is due to recent transmission (India) and slower where more cases arise from the reactivation of latent infection (Korea) [32], [34]. For either India or Korea, the comparatively weak adverse effects identified here ought to be surmountable by early case detection and treatment, though the challenge is greater for India.

If such improvements in chemotherapy are not made there is a risk, especially in India, that nutritional and demographic changes will generate more TB cases per capita. The risk in future is greater than portrayed by our results because the pace of aging and urbanization in India is accelerating [17]. For example, the proportion of India's population living in urban areas increased from 13% in 1990 to 15% in 2010, but is expected to reach 20% by 2030 [17]. The average age of the Indian population increased by 3 years between 1990 and 2010 (24.9 to 27.9 years); it will increase by a further 5 years by 2030 (to 32.9 years). In theory, any process that increases incidence per capita will also increase the risk of infection through transmission, leading to a further increase in case load [32]. This positive feedback process generates exponential growth in case load, at least in the short term, a significant penalty for relaxing control by chemotherapy.

Even if TB control programmes have no direct influence over nutritional and demographic changes in populations they can, at a minimum, prevent their worst consequences. Better still, TB control will be supported by the prevention and active management of various forms of malnutrition (including diabetes and under-nourishment), adding to the decline in TB incidence. By aligning all positive factors, both India and Korea will be in a stronger position to maintain the decline of TB in their growing, aging, urbanizing populations.

The present analysis is limited in four main respects. First, the data from India and Korea come from a combination of more and less extensive surveys, carried out by different methods, gathering data of variable quality. For example, diabetes prevalence (1999–2002) and BMI data (1998, 2005) from India were drawn from different surveys done at different times, which had to be combined (Text S1). Second, our analysis is restricted to India and Korea, because these appear to be the only two countries at present that can provide enough data to examine the principal interactions among this combination of risk factors. Third, the structure of model (1) allows for known interactions among risk factors; we do not exclude the possibility that others exist, although it seems unlikely that there are powerful but as yet unknown effects. Finally, our deduction that early TB case detection and treatment can overcome the adverse effects of the various risk factors investigated here merits a fuller investigation – in theory, in practice, and for a wider range of countries.

## Supporting Information

Text S1 Nutrition, diabetes and tuberculosis in the epidemiological transition.
(DOC)
