# Supplementary material for: Nutrition, Diabetes and Tuberculosis in the Epidemiological Transition
Source: PLoS One. 2011 Jun 21;6(6):e21161. doi: 10.1371/journal.pone.0021161 (PMC3119681; doi:10.1371/journal.pone.0021161)
Supplement: Text S1 — Nutrition, diabetes and tuberculosis in the epidemiological transition. (DOC) [file pone.0021161.s001.doc]

**Nutrition, diabetes and tuberculosis in the epidemiological transition (supporting information)**

**Text S1**

**Christopher Dye^1^, Bernadette Bourdin Trunz^2^, Knut Lönnroth^3^, Gojka Roglic^4^, Brian G Williams**

This appendix gives further details of the sources of data and how they have been used in the analysis described in the main paper.

**Populations**. Populations by age and sex, and in urban and rural areas, by year, are point estimates calculated by the UN Population Division, without any measure of uncertainty [1,2]. The errors surrounding these estimates, especially errors in the rate of change of population size and age structure over time, are likely to be small compared with the other sources of uncertainty that we did allow for in this analysis.

**Body Mass Index**. Expanding on the data presented in the main paper, Figures S1 (India) and S2 (Republic of Korea) show the distributions by BMI group of men and women, in urban and rural areas, and in initial and final years. The data in Figures S1 and S2 were obtained by combining survey data giving the proportion of people in each of the BMI groups used for India [3,4] and Korea [5,6,7] with the national population sizes of each of those groups. Sample sizes in the original survey reports for India and Korea allow calculation of standard deviations of the proportions of people in each BMI group, *i* (age, sex, rural/urban, year). These are the standard deviations used in uncertainty analysis.

In using function $f(b)=e^{-a(b-b_{\min})}$ to define the relationship between BMI (*b*) and per capita TB incidence in model (1) we assumed that any increase in BMI is more protective against TB. However, given uncertainties in the original data [8], it is possible that the risk of TB, like diabetes, increases among obese people (i.e. the minority with BMI > 30 kg/m^2^), although the numbers show no statistically significant departure from exponential decline. If the risk of TB does increase at high BMI, that would weaken the protective effect of rising BMI overall. In the absence of any further evidence, we have retained a simple exponential form for *f*(*b*).

**Diabetes.** For Korea, all information about diabetes prevalence in relation to BMI, age and sex comes from KNHANES surveys, which used American Diabetes Association (ADA) diagnostic criteria [5,6,7]. A 2006 survey in the south-east of the country also reported differences between urban and rural areas [9]. For India, by contrast, diabetes prevalence (1999–2002) [10] and BMI (1998, 2005) [3,11] come from different surveys, which need to be combined. The national PODIS survey carried out in India between 1999 and 2002 (median year 2001) – a random multistage cross-sectional population survey – reported diabetes prevalence by BMI, age, sex and in urban and rural areas. Diabetes diagnosis based on WHO [10] and ADA [12] criteria in PODIS gave similar results (sample prevalences of 4.3% and 3.3%, respectively), albeit in surveys that were not precisely comparable. We used results obtained by the WHO criteria.

Figure S3A (filled bars) shows diabetes prevalence in relation to BMI, with people ≥25kg/m^2^ combined in the highest BMI group (SM Sadikot, pers. comm.). To a good approximation, diabetes prevalence increased linearly with BMI (open bars), and we used linear regression to calculate the relative diabetes prevalence (with standard deviations) in the three BMI groups 18.5–24.9, 25.0–29.9 and ≥30kg/m^2^ compared with the lowest group(<18.5 kg/m^2^, Figure S3B). Diabetes prevalence increased more steeply with BMI in India than in Korea (Figure S3B), but Korea had a higher prevalence on average (Figures 1 and 2 of main text).

For India, PODIS also reported diabetes prevalence by age, sex and in urban and rural areas. The increase in prevalence with age was approximately linear, and analysis of covariance showed that the slope of linear regression did not differ significantly between urban and rural areas (Figure S4).There was also no significant difference in prevalence between men and women (4.4% and 4.5%, respectively, in adults ≥25 years old). Using these data, we calculated the prevalence in the two age groups adopted for India, 15–49 and ≥50 years, standardizing for population age structure, and we took prevalence in urban areas to be an additional 2.44% (sd 0.64%) higher than in rural areas in each age group. We assumed equal prevalence in men and women. These results are consistent with the findings of two other surveys carried out in India in 2000 (stratified random sampling, confined to urban areas) [13] and 2002 (self-reported diabetes in selected urban and rural centres) [14], which found that prevalence increased with BMI and age, was higher in urban than rural areas, and did not differ between men and women.

The national prevalence of diabetes in India in 1998 and 2005 was also derived from measurements given in PODIS, projecting backwards from median year 2001 to 1998 and forwards to 2005 using the only trend data available for India (from Chennai) [15]. Although there is much uncertainty about diabetes trends in India, it is clear that (a) prevalence is increasing, and (b) increasing faster than can be explained simply by population aging and urbanization [16,17]. With our interpretation of Indian data, changes in BMI, population age structure and urbanization account for an increase in prevalence among people aged ≥15 years from 3.0% to 3.6% between 1998 and 2005, whereas trends derived from the Chennai data take prevalence up to 3.7% in 2005. Our assessment of the overall increase in national prevalence is likely to be conservative.

Our analysis for Korea drew on data from the first and last (of five) KNHANES surveys in 1998 and 2008 [5,6]. Figure S5 shows that the difference in diabetes prevalence between these two years was consistent with the trend over the whole period.

**Tuberculosis.** By assuming that all TB cases occur in adults, we overestimated the per capita incidence rate in this group (because a minority of cases are in children). However, this assumption does not obscure the very large (twofold) difference in per capita incidence rate between Indian and Korean populations. Furthermore, we are mostly concerned here, not with absolute estimates but with the change in case numbers and rates through time.

Figures S6 and S7 show the reported smear-positive incidence rates by age and sex, respectively, scaled to the estimated total rate for all forms of TB (filled bars) [18], compared with model calculations (open bars). Although the reported *relative* incidence rates among age groups (used to calculate *R_a_*) and between men and women (used to calculate *R_s_*) could be biased, we assumed that these relative incidence rates have negligible random errors, and therefore did not contribute to uncertainty in outcomes.

The key output variables are the 10-year increases in TB cases (absolute numbers and per capita) and in TB cases among people with diabetes. These figures are presented with 95% confidence limits.

**Uncertainty analysis.** Throughout the study, uncertainty analysis was carried out with *@Risk* (Palisade Corporation). Variation was assumed to be normally distributed with standard deviations determined from the data described above. Errors derived by simulation are a Monte Carlo estimate of 95% confidence limits.

Figure S1. Population distribution of BMI in India in 1998-99 (25-64 years, taken to be for 1998) and 2005-06 (15-49 years, taken to be for 2005). On average, BMI grew between the two years, but there was a noticeable increase through time in the proportion of men with low BMI (<18.5 kg/m^2^) in rural areas (bottom left).

Figure S2. Population distribution of BMI in Korea in men and women in urban and rural areas, in 1998 and 2008. Average BMI increased between the two years in all four groups.

Figure S3. Diabetes prevalence in India (A) as reported in the PODIS survey, 1999–2002 (filled bars, average for men and women) showing the approximately linear increase with BMI (open bars) [10]. The highest BMI group contains all people ≥25kg/m^2^. (B) The linear model was used to calculate relative diabetes prevalence in four BMI groups <18.5, 18.5–24.9, 25.0–29.9 and ≥30kg/m^2^. The relative prevalence of diabetes is shown for both India (filled circles) and Korea (open circles).

Figure S4. Diabetes prevalence by age in urban (filled circles) and rural (open circles) areas of India, as reported in the PODIS survey, 1999-2002. The increase in diabetes prevalence with age was not significantly different from linear, and the slope of a linear regression (0.131/year, sd 0.024/year) did not differ between urban (upper line) and rural (lower line) areas. In urban areas, diabetes prevalence was 2.44% (sd 0.636%) higher than in rural areas at any given age. Error bars are standard deviations based on survey sample sizes.

Figure S5. Prevalence of diabetes among men (filled bars) and women (open bars) in Korea in all five KNHANES surveys.

Figure S6. TB incidence/100,000/year by age group in (A, B) India and (C, D) Korea in initial (1998) and final (2005 or 2008) years. Black bars are reported smear-positive incidence rates scaled to the estimated total rate for all forms of TB; white bars are model calculations.

Figure S7. TB incidence/100,000/year by sex in (A, B) India and (C, D) Korea in initial (1998) and final (2005 or 2008) years. Black bars are reported smear-positive incidence rates scaled to the estimated total rate for all forms of TB; white bars are model calculations.

**References**

1. United Nations DoEaSA, Population Division (2009) World Population Prospects: The 2008 Revision. New York City: United Nations.

2. United Nations DoEaSA, Population Division (2010) World Urbanization Prospects: The 2009 Revision. New York City: United Nations.

3. International Institute for Population Sciences (IIPS) and ORC Macro (2000) National Family Health Survey (NFHS-2), 1998-1999: India. Mumbai: IIPS.

4. International Institute for Population Sciences (IIPS) and Macro International (2007) National Family Health Survey (NFHS-3), 2005-06, India: Key Findings. Mumbai: IIPS.

5. Kim Y, Suh YK, Choi H (2004) BMI and metabolic disorders in South Korean adults: 1998 Korea National Health and Nutrition Survey. Obesity Research 12: 445-453.

6. Choi YJ, Kim HC, Kim HM, Park SW, Kim J, et al. (2009) Prevalence and management of diabetes in Korean adults: Korea National Health and Nutrition Examination Surveys 1998-2005. Diabetes Care 32: 2016-2020.

7. Kim WY, Kim JE, Choi YJ, Huh KB (2008) Nutritional risk and metabolic syndrome in Korean type 2 diabetes mellitus. Asia Pacific Journal of Clinical Nutrition 17 Suppl 1: 47-51.

8. Lönnroth K, Williams BG, Dye C (2010) A consistent log-linear relationship between tuberculosis incidence and body-mass index. International Journal of Epidemiology 39: 149-155.

9. Lee HY, Won JC, Kang YJ, Yoon SH, Choi EO, et al. (2010) Type 2 diabetes in urban and rural districts in Korea: factors associated with prevalence difference. Journal of Korean Medical Science 25: 1777-1783.

10. Sadikot SM, Nigam A, Das S, Bajaj S, Zargar AH, et al. (2004) The burden of diabetes and impaired glucose tolerance in India using the WHO 1999 criteria: prevalence of diabetes in India study (PODIS). Diabetes Research and Clinical Practice 66: 301-307.

11. International Institute for Population Sciences (IIPS) and Macro International (2007) National Family Health Survey (NFHS-3), 2005-06, India. Mumbai: IIPS.

12. Sadikot SM, Nigam A, Das S, Bajaj S, Zargar AH, et al. (2004) The burden of diabetes and impaired fasting glucose in India using the ADA 1997 criteria: prevalence of diabetes in India study (PODIS). Diabetes Research and Clinical Practice 66: 293-300.

13. Ramachandran A, Snehalatha C, Kapur A, Vijay V, Mohan V, et al. (2001) High prevalence of diabetes and impaired glucose tolerance in India: National Urban Diabetes Survey. Diabetologia 44: 1094-1101.

14. Mohan V, Mathur P, Deepa R, Deepa M, Shukla DK, et al. (2008) Urban rural differences in prevalence of self-reported diabetes in India–the WHO–ICMR Indian NCD risk factor surveillance. Diabetes Research and Clinical Practice 80: 159-168.

15. Mohan V, Deepa M, Deepa R, Shanthirani CS, Farooq S, et al. (2006) Secular trends in the prevalence of diabetes and impaired glucose tolerance in urban South India–the Chennai Urban Rural Epidemiology Study (CURES-17). Diabetologia 49: 1175-1178.

16. Colagiuri S, Borch-Johnsen K, Glümer C, Vistisen D (2005) There really is an epidemic of type 2 diabetes. Diabetologia 48: 1459-1463.

17. Gupta A, Misra A (2007) Review: Type 2 diabetes in India: regional disparities. The British Journal of Diabetes & Vascular Disease 7: 12-16.

18. World Health Organization (2010) Global Tuberculosis Control 2010. Geneva: World Health Organization.
